# Supplementary material for: Netrin Family Genes as Prognostic Markers and Therapeutic Targets for Clear Cell Renal Cell Carcinoma: Netrin-4 Acts through the Wnt/β-Catenin Signaling Pathway
Source: Cancers (Basel). 2023 May 18;15(10):2816. doi: 10.3390/cancers15102816 (PMC10216638; doi:10.3390/cancers15102816)
Supplement: Supplementary file 1 [file cancers-15-02816-s001.zip › Supplementary Figure S1 The blots of all bands and molecular weight markers.pdf]

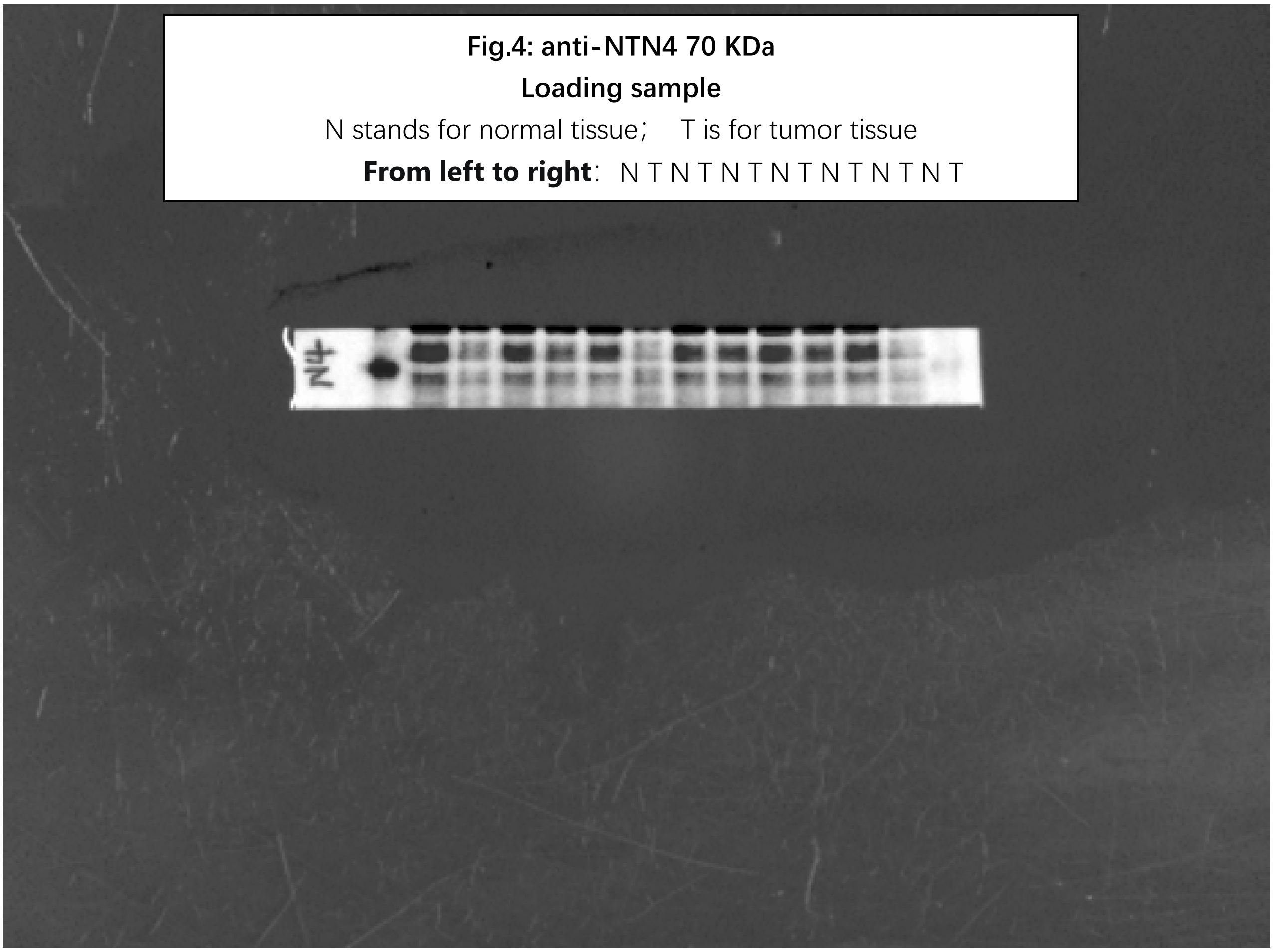

Fig.4: anti-NTN4 70 KDa

Loading sample

N stands for normal tissue; T is for tumor tissue

From left to right: N T N T N T N T N T N T N T

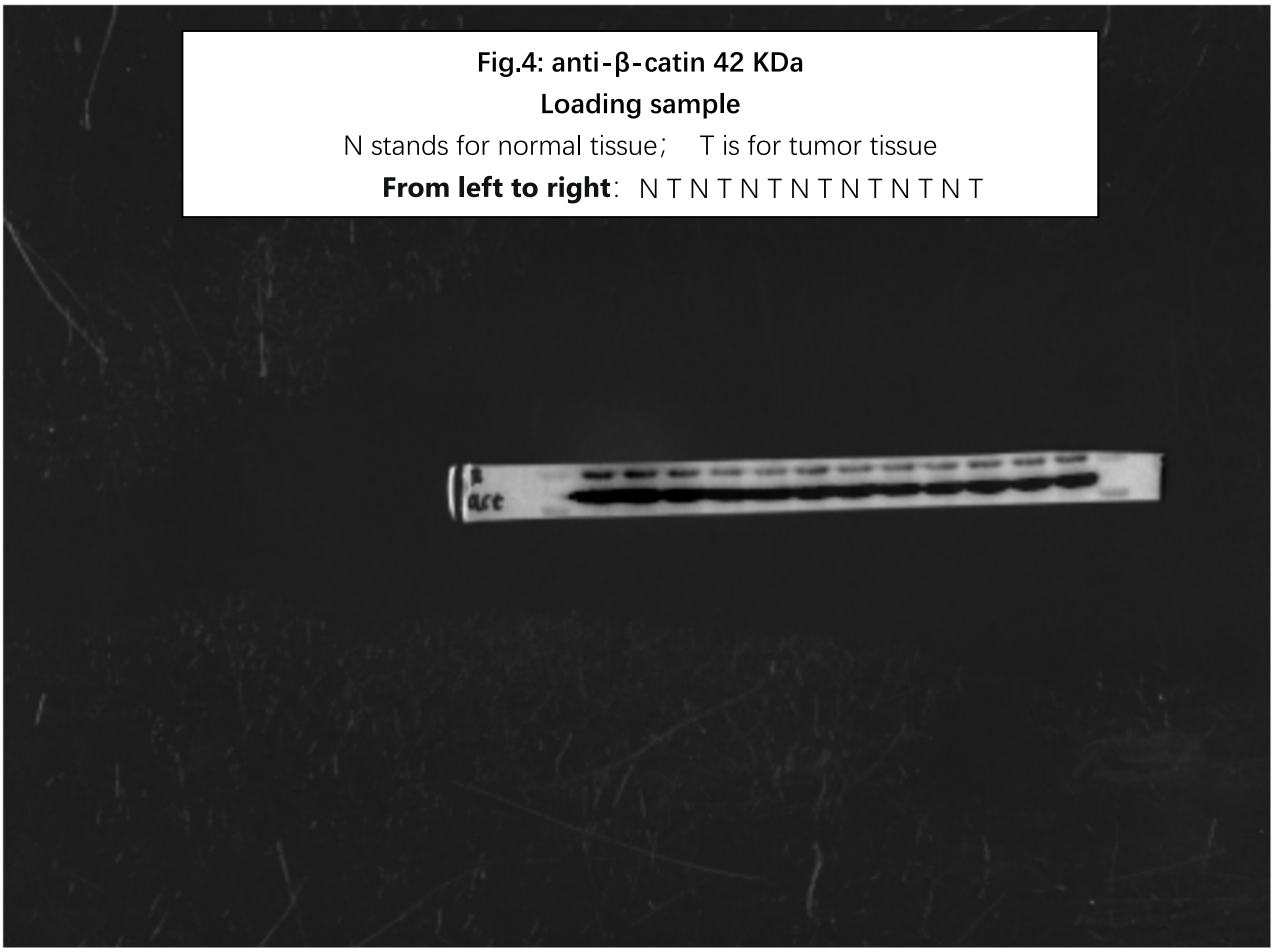

Fig.4: anti-β-catin 42 KDa

Loading sample

N stands for normal tissue; T is for tumor tissue

From left to right: N T N T N T N T N T N T N T

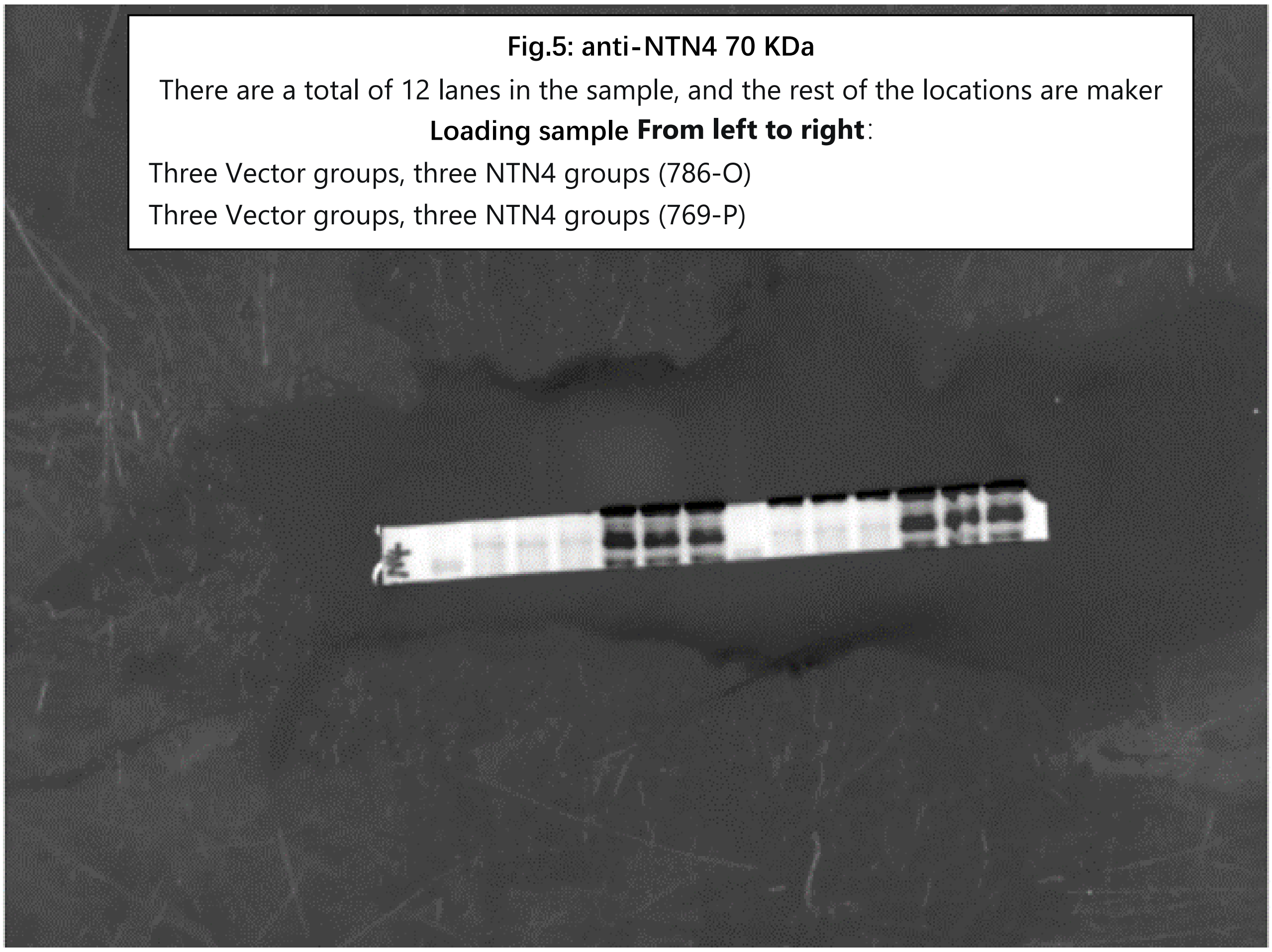

Fig.5: anti-NTN4 70 KDa

There are a total of 12 lanes in the sample, and the rest of the locations are maker

Loading sample From left to right:

Three Vector groups, three NTN4 groups (786-O)

Three Vector groups, three NTN4 groups (769-P)

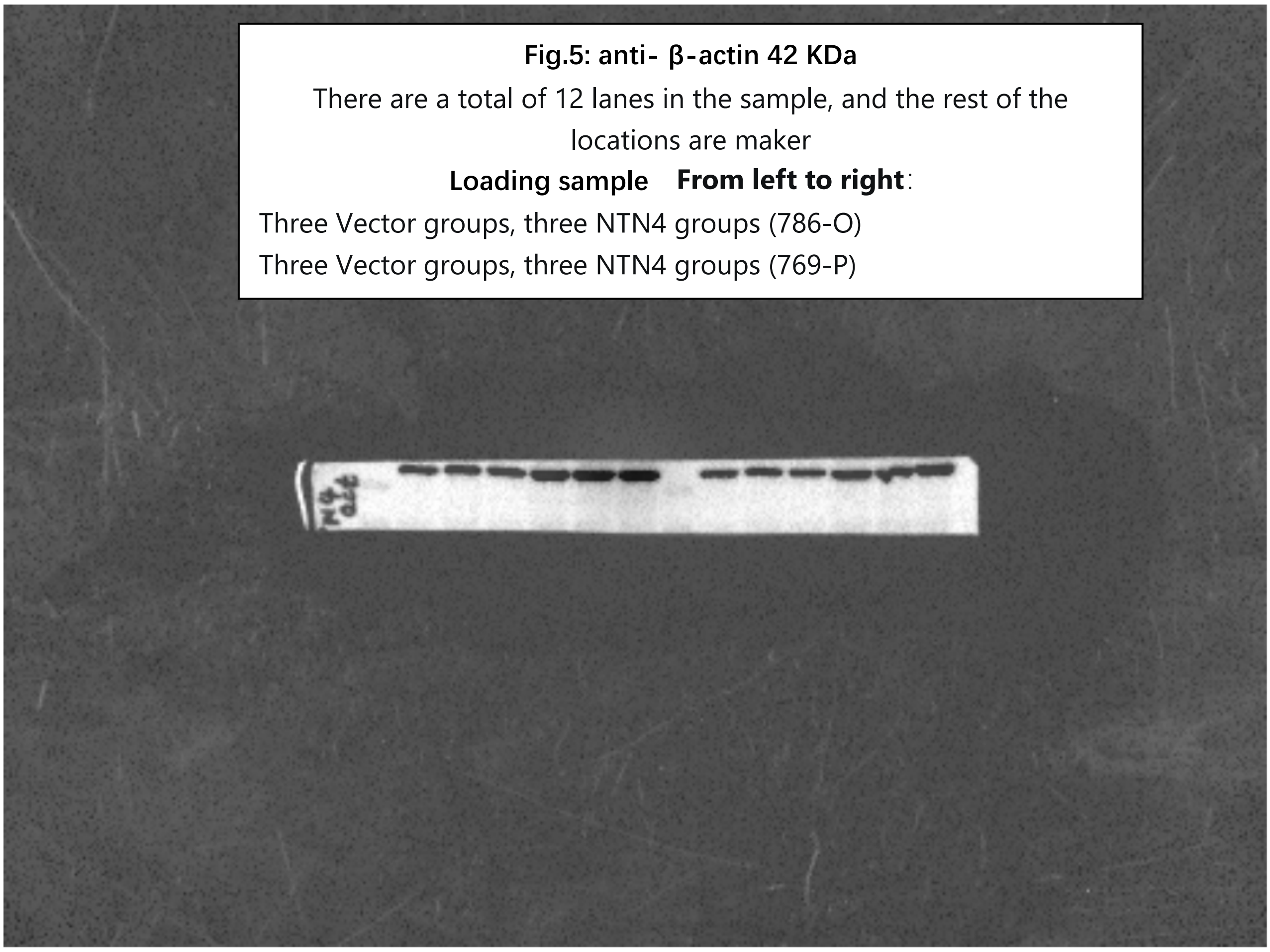

Fig.5: anti- β-actin 42 KDa

There are a total of 12 lanes in the sample, and the rest of the locations are maker

Loading sample From left to right:

Three Vector groups, three NTN4 groups (786-O)

Three Vector groups, three NTN4 groups (769-P)

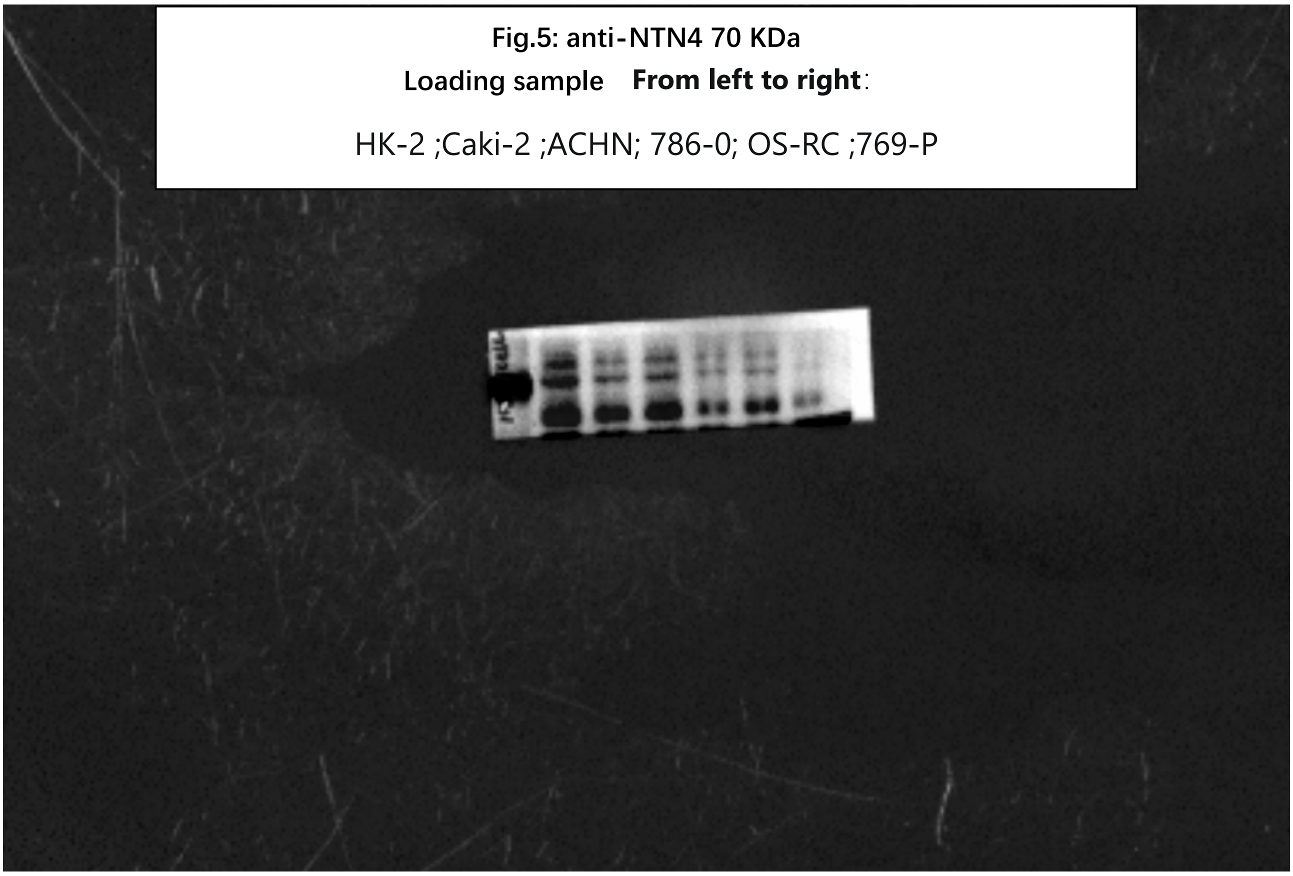
Fig.5: anti-NTN4 70 KDa
Loading sample From left to right:
HK-2 ;Caki-2 ;ACHN; 786-0; OS-RC ;769-P

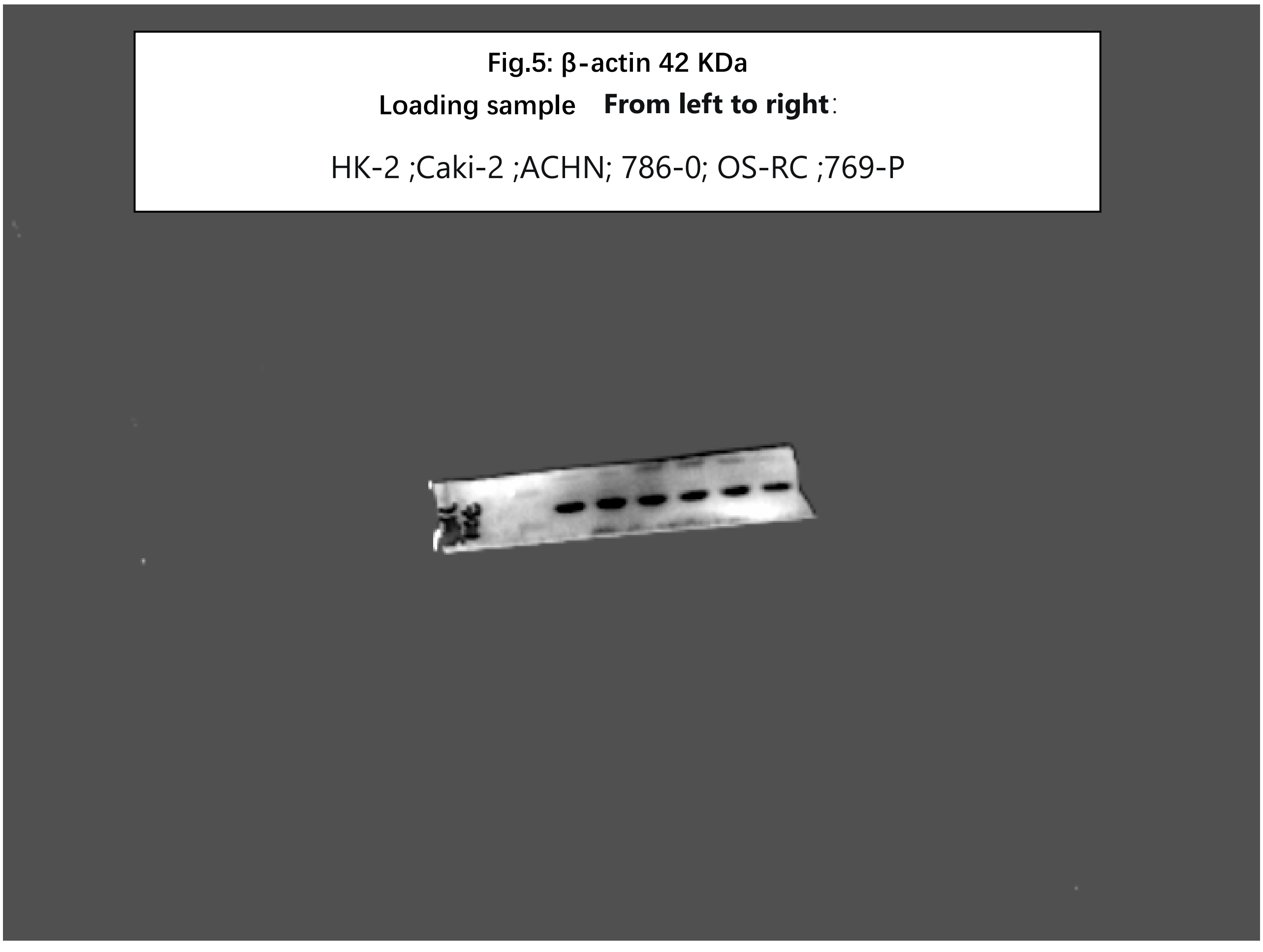
Fig.5: β-actin 42 KDa
Loading sample From left to right:
HK-2 ;Caki-2 ;ACHN; 786-0; OS-RC ;769-P

Fig.6: anti-E-CAD 120 KDa

There are a total of 12 lanes in the sample, and the rest of the locations are maker

Loading sample From left to right:

Three Vector groups, three NTN4 groups (786-O)

Three Vector groups, three NTN4 groups (769-P)

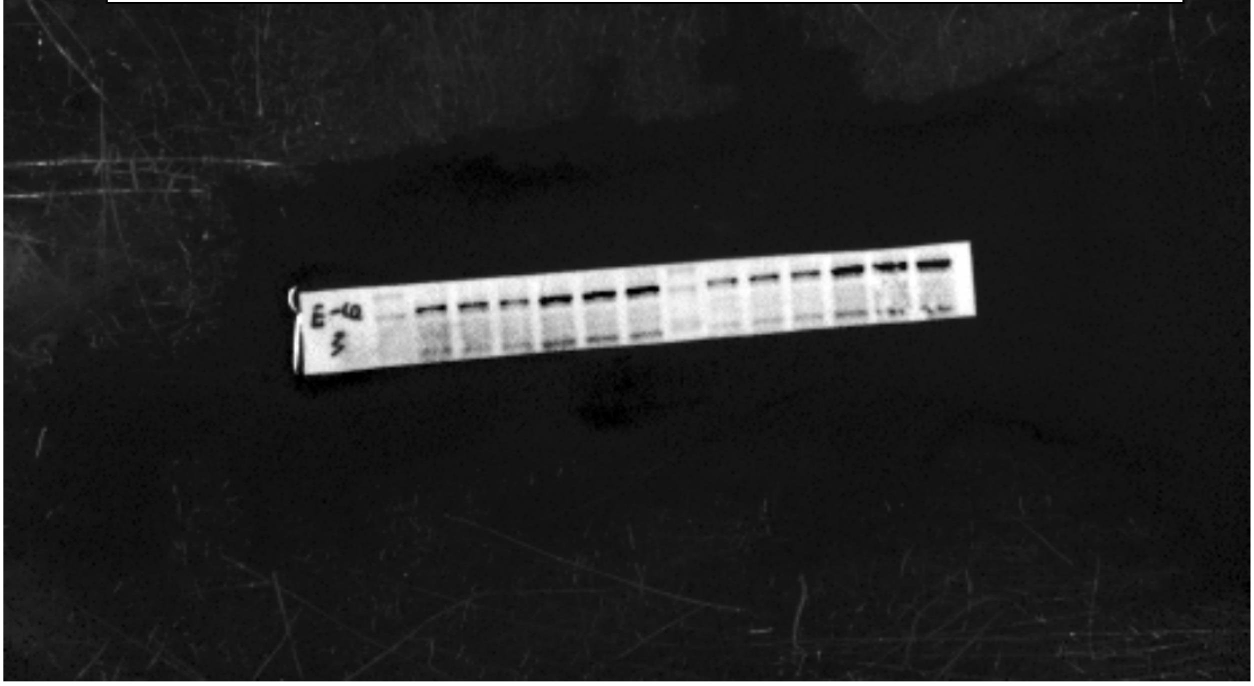

Fig.6: anti-N-CAD 130 KDa

Loading sample From left to right:

Three Vector groups, three NTN4 groups (786-O)

Three Vector groups, three NTN4 groups (769-P)

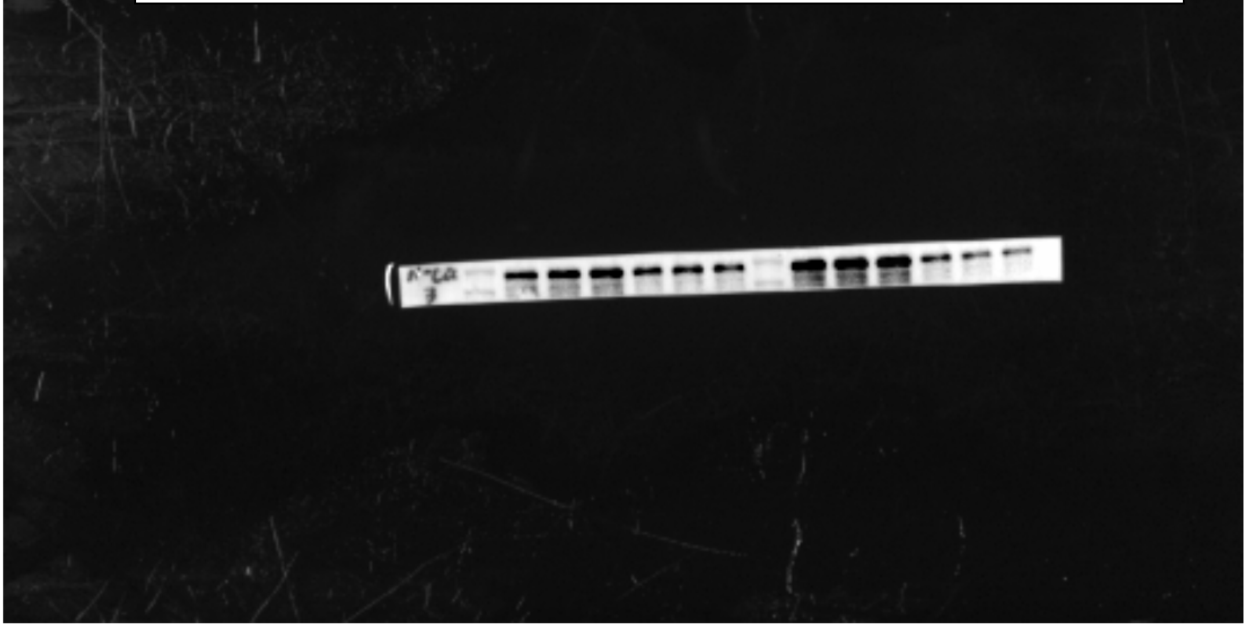

\

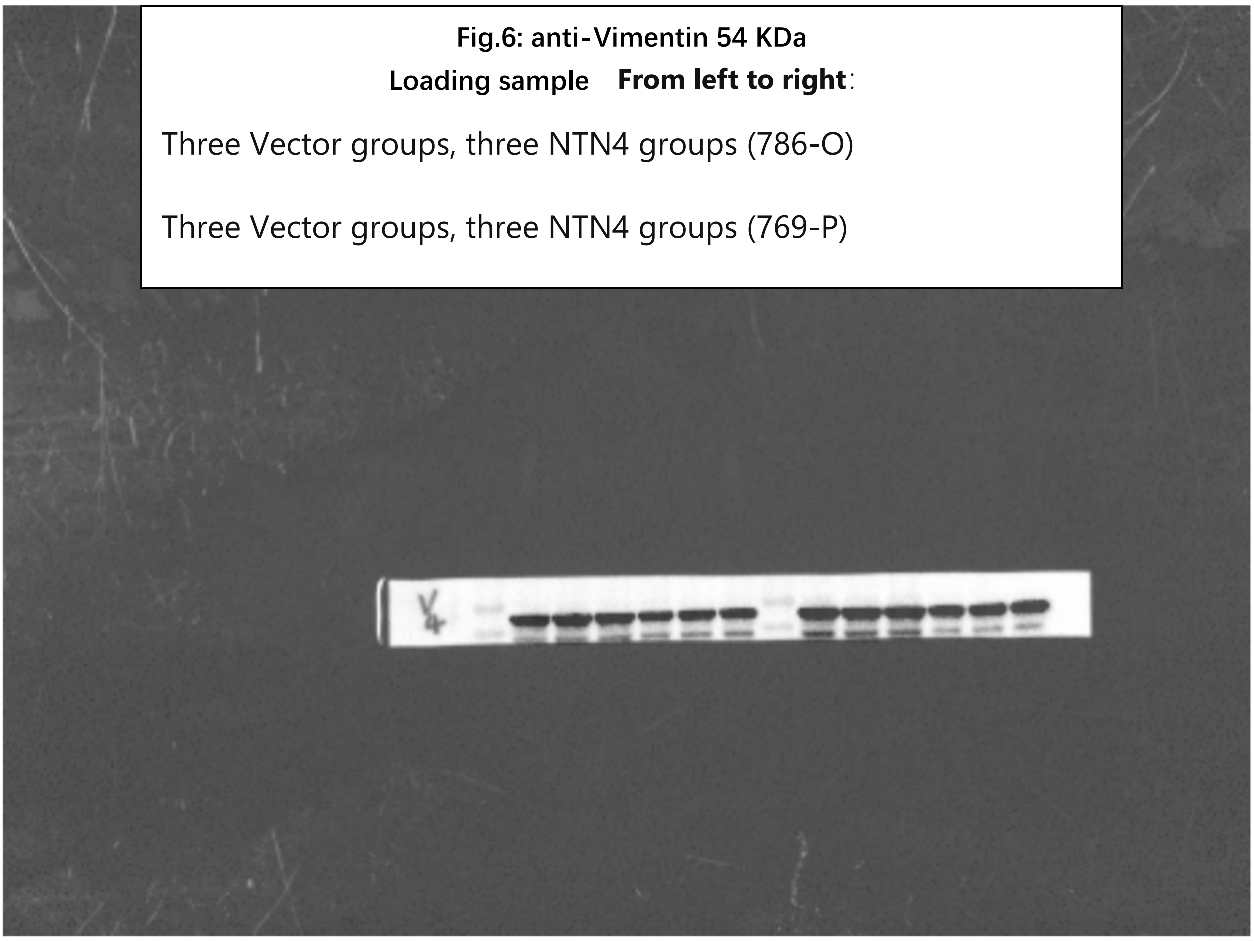

Fig.6: anti-Vimentin 54 KDa

Loading sample From left to right:

Three Vector groups, three NTN4 groups (786-O)

Three Vector groups, three NTN4 groups (769-P)

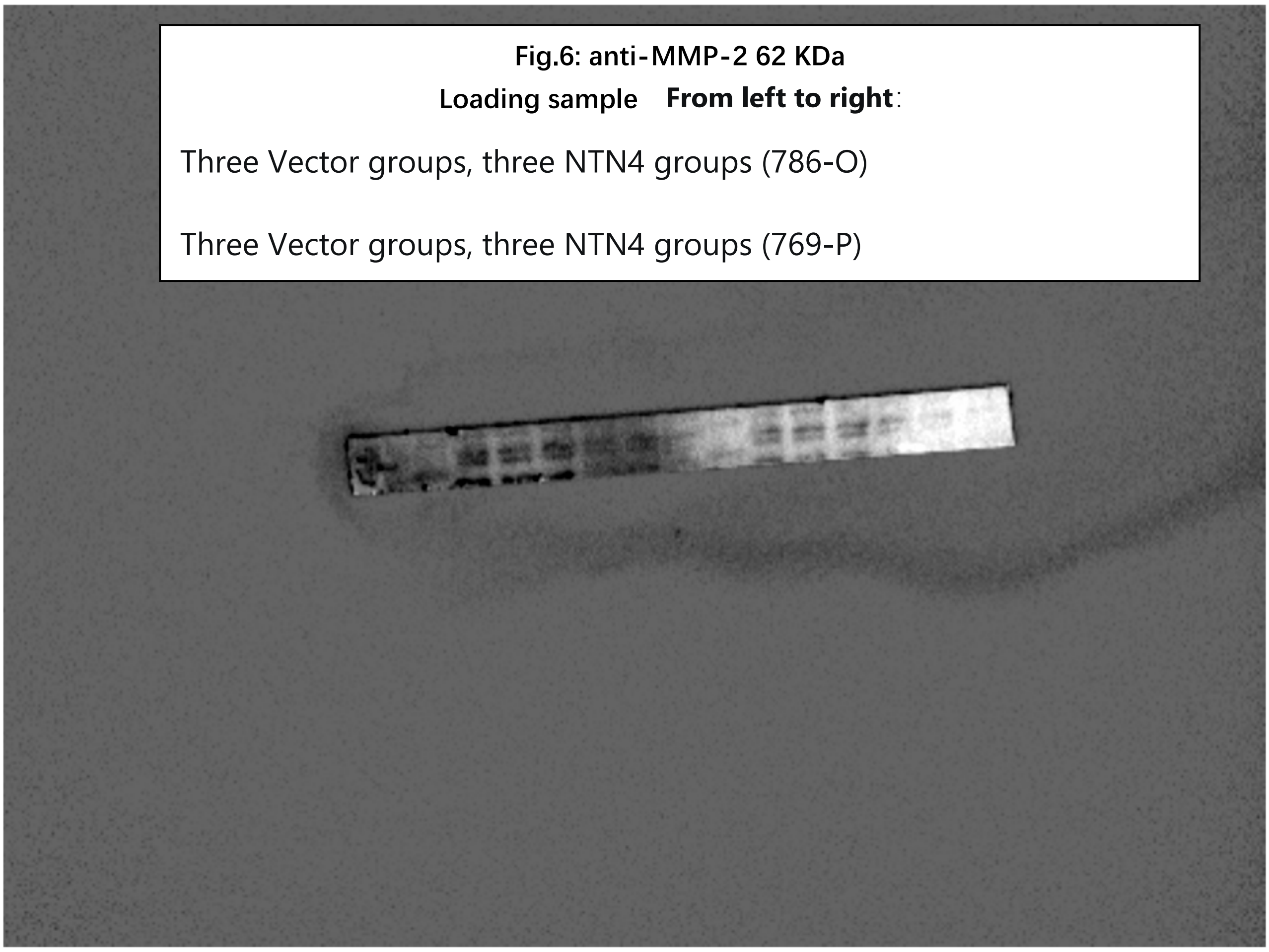

Fig.6: anti-MMP-2 62 KDa

Loading sample From left to right:

Three Vector groups, three NTN4 groups (786-O)

Three Vector groups, three NTN4 groups (769-P)

Fig.6: β-actin 42 KDa

Loading sample From left to right:

Three Vector groups, three NTN4 groups (786-O)

Three Vector groups, three NTN4 groups (769-P)

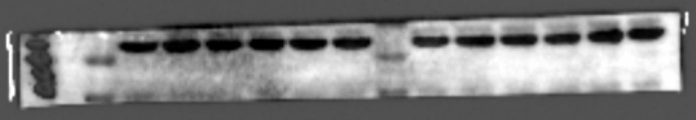

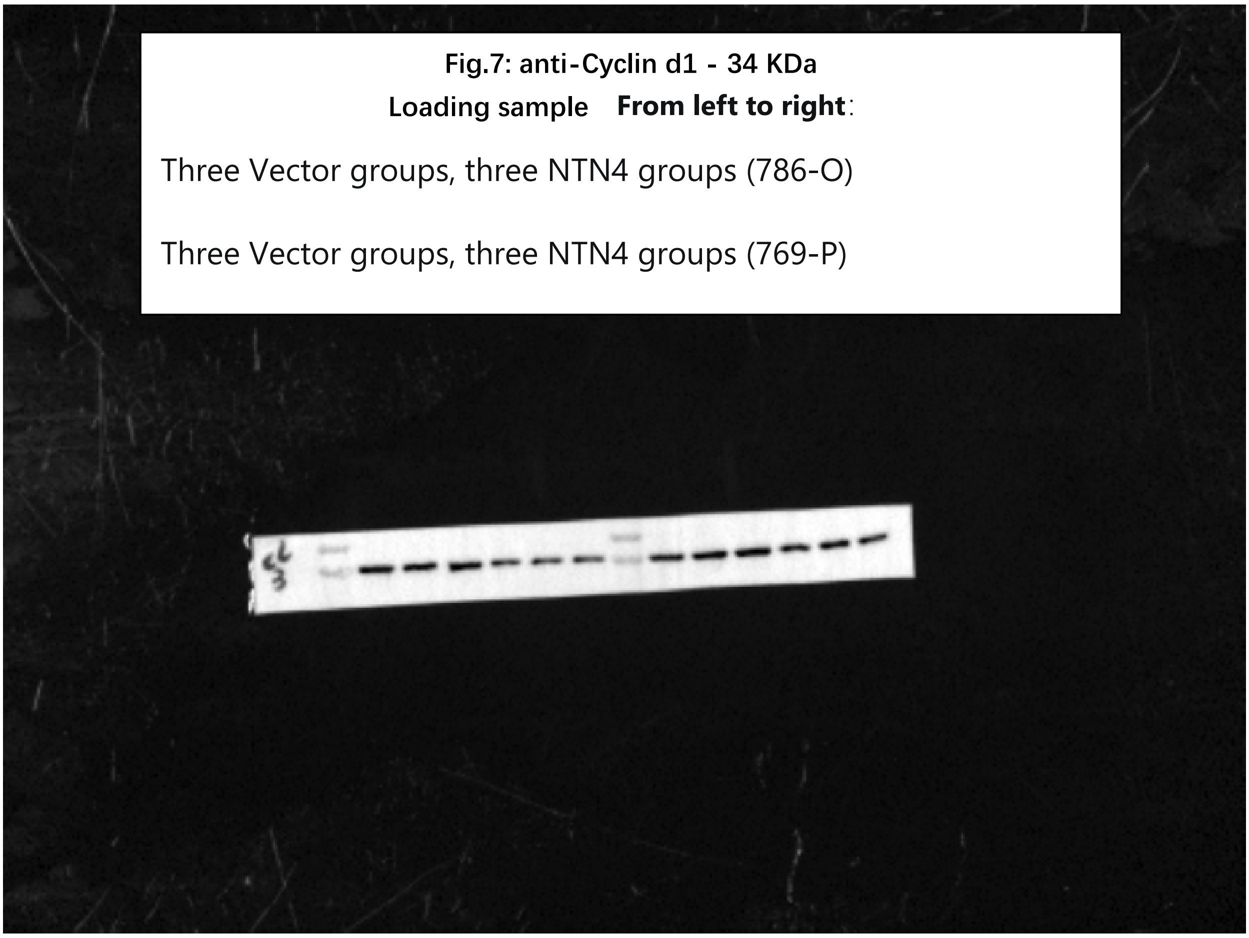
Fig.7: anti-Cyclin d1 - 34 KDa
Loading sample From left to right:
Three Vector groups, three NTN4 groups (786-O)
Three Vector groups, three NTN4 groups (769-P)

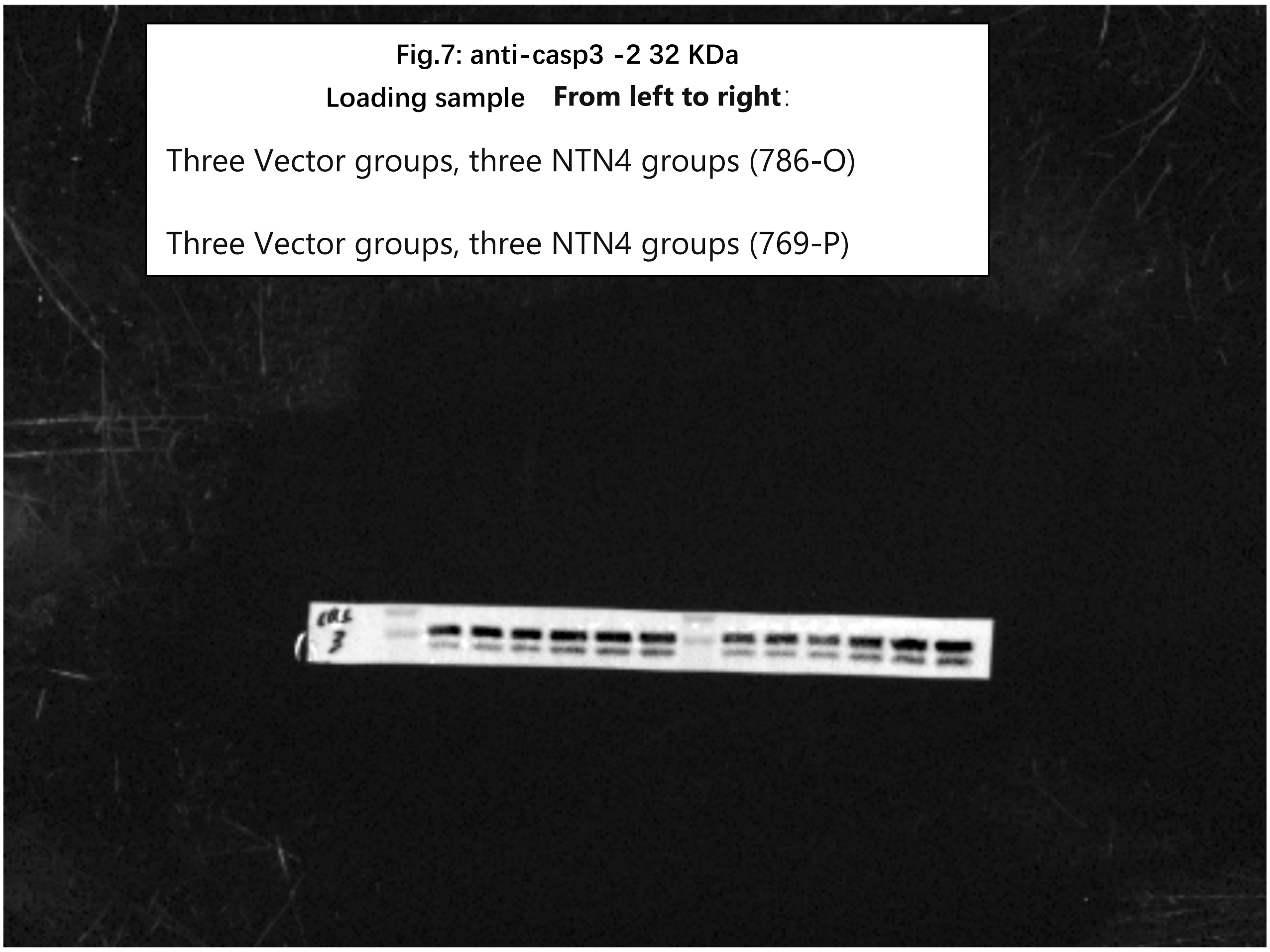
Fig.7: anti-casp3 -2 32 KDa
Loading sample From left to right:
Three Vector groups, three NTN4 groups (786-O)
Three Vector groups, three NTN4 groups (769-P)

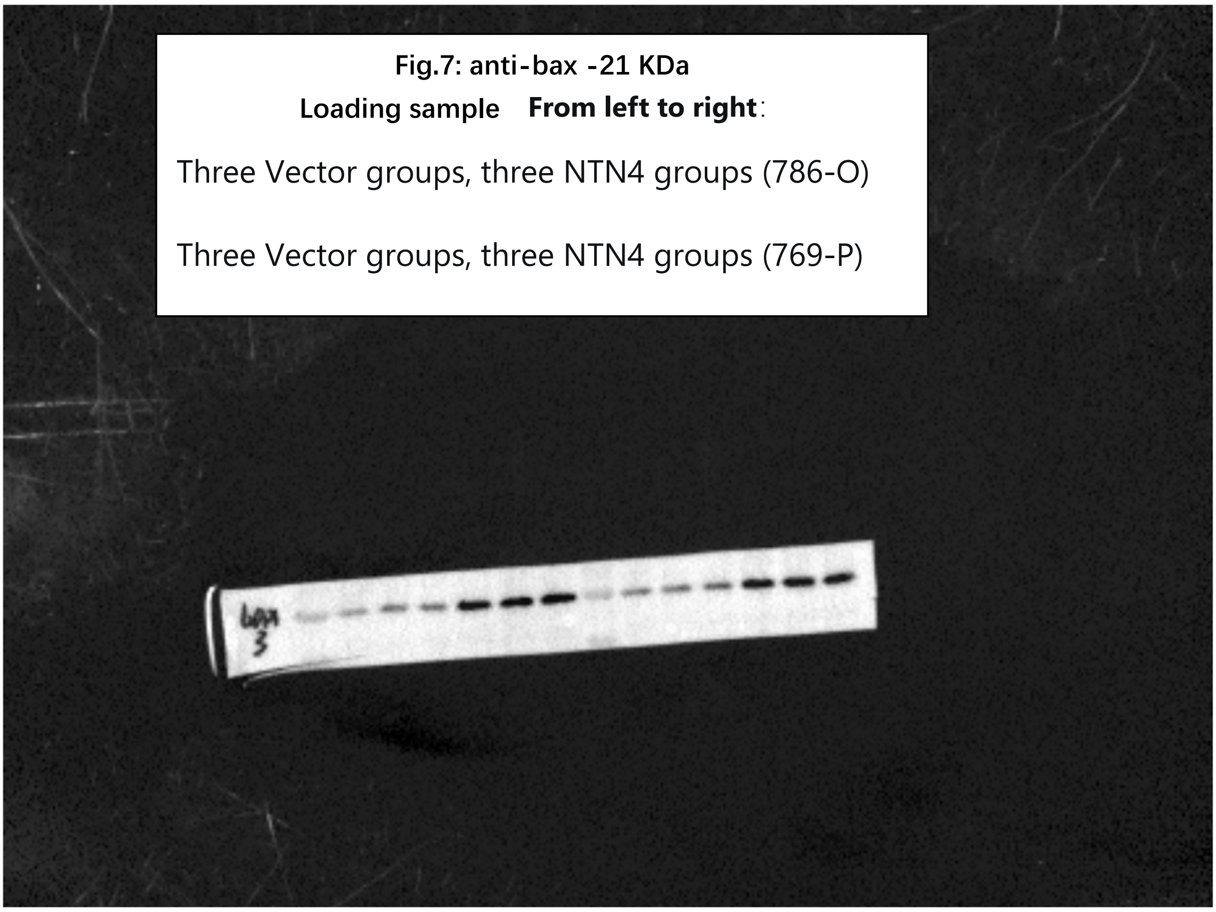
Fig.7: anti-bax -21 KDa
Loading sample From left to right:
Three Vector groups, three NTN4 groups (786-O)
Three Vector groups, three NTN4 groups (769-P)

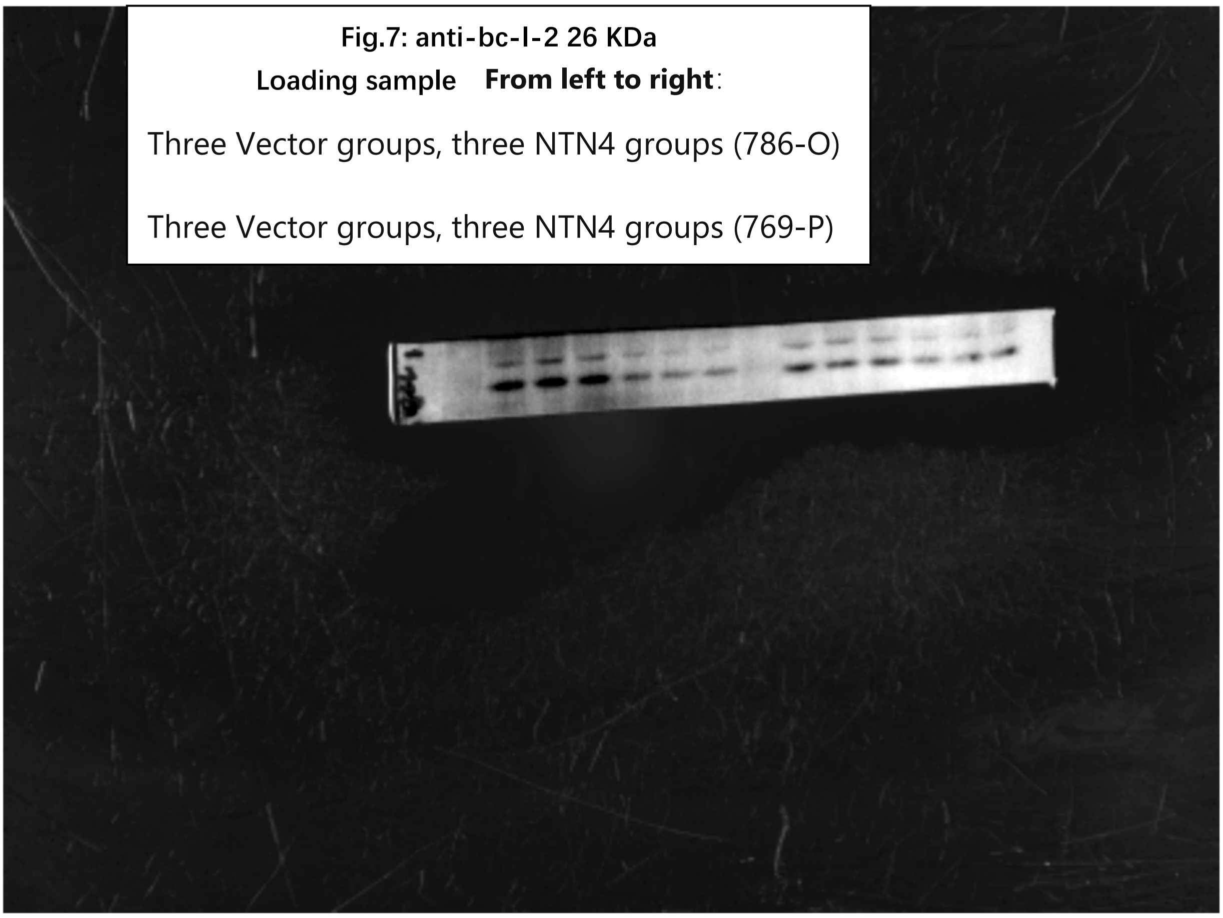
Fig.7: anti-bc-l-2 26 KDa
Loading sample From left to right:
Three Vector groups, three NTN4 groups (786-O)
Three Vector groups, three NTN4 groups (769-P)

Fig.7: anti-β-actin 42 KDa

Loading sample From left to right:

Three Vector groups, three NTN4 groups (786-O)

Three Vector groups, three NTN4 groups (769-P)

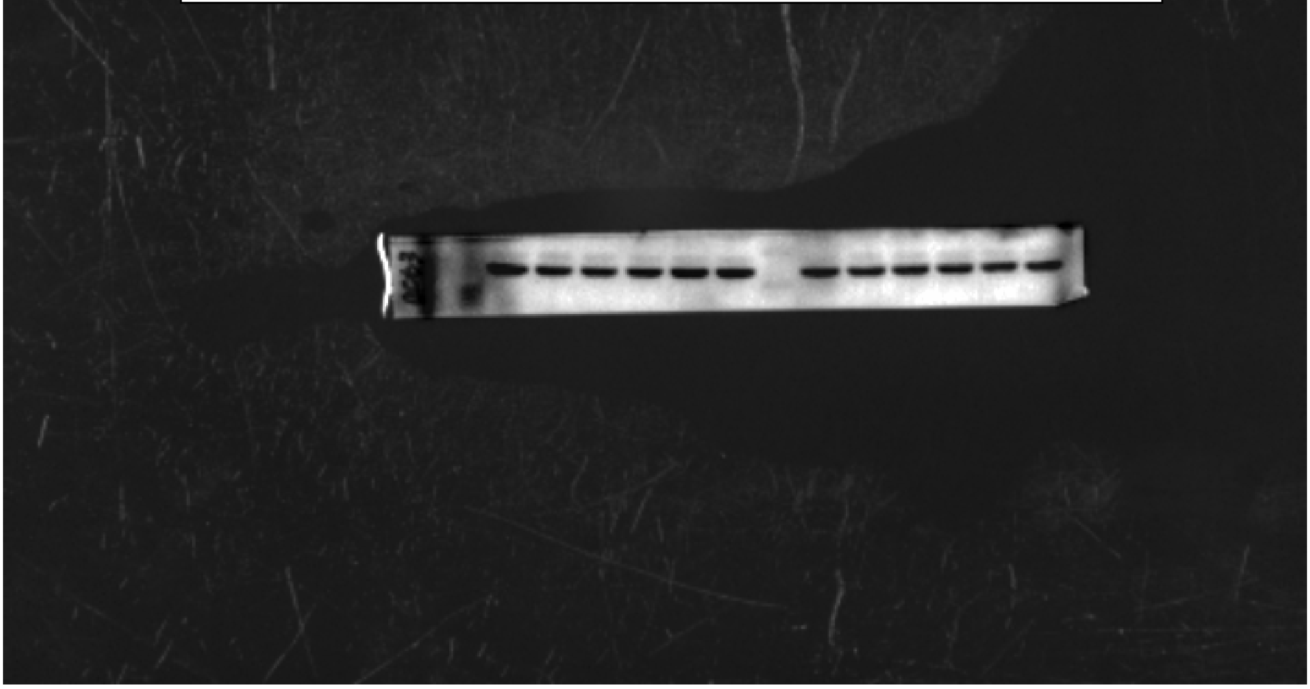

Fig.8: anti-p-GSK3β 48 KDa

Loading sample From left to right:

Three Vector groups, three NTN4 groups (786-O)

Three Vector groups, three NTN4 groups (769-P)

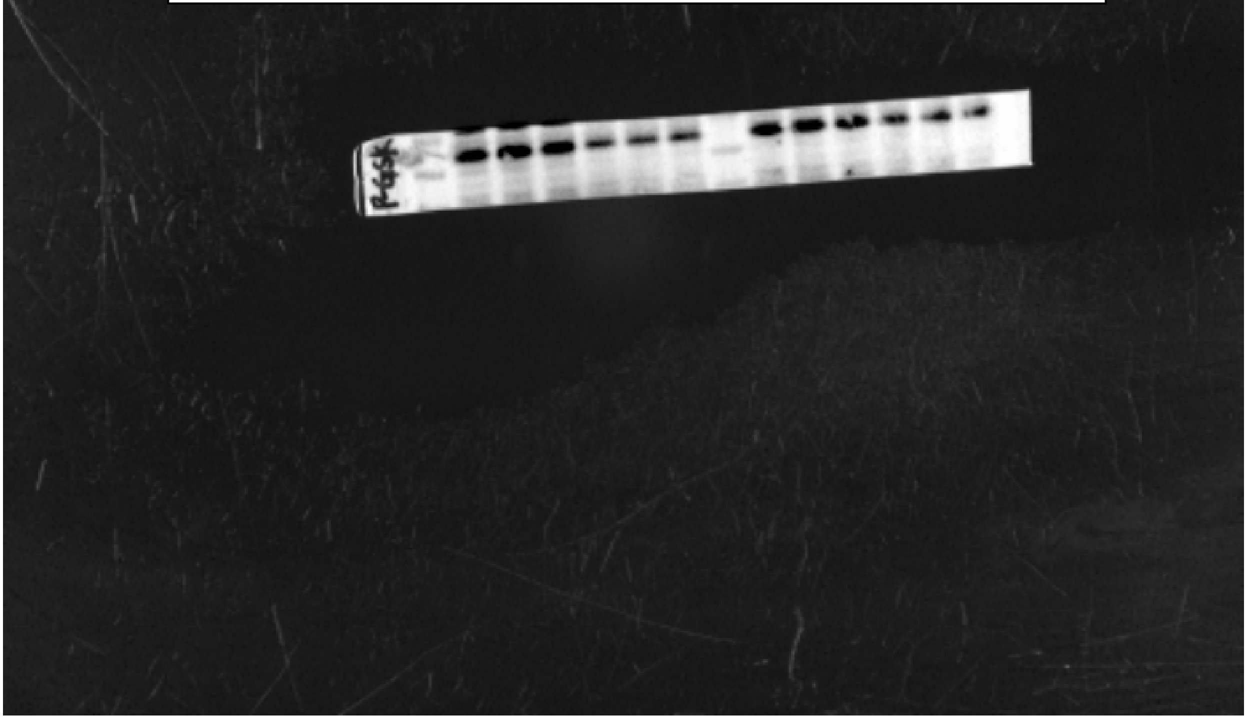

Fig.8: anti- GSK3β 48 KDa

Loading sample From left to right:

Three Vector groups, three NTN4 groups (786-O)

Three Vector groups, three NTN4 groups (769-P)

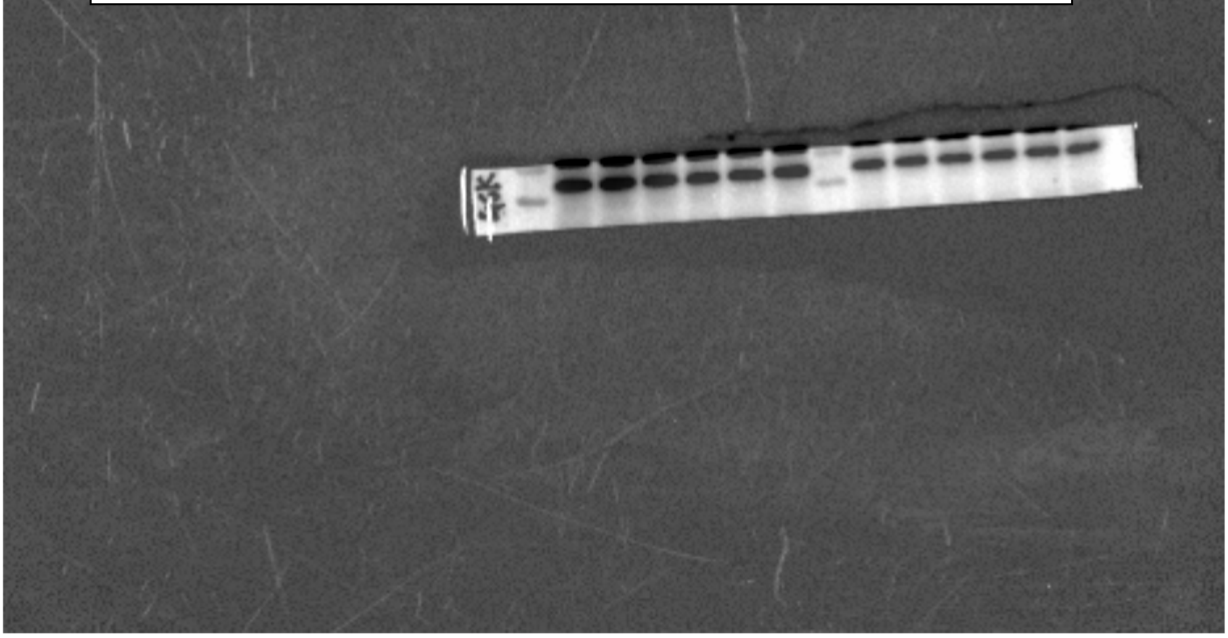

Fig.8: anti-β-catenin 92 KDa

Loading sample From left to right:

Three Vector groups, three NTN4 groups (786-O)

Three Vector groups, three NTN4 groups (769-P)

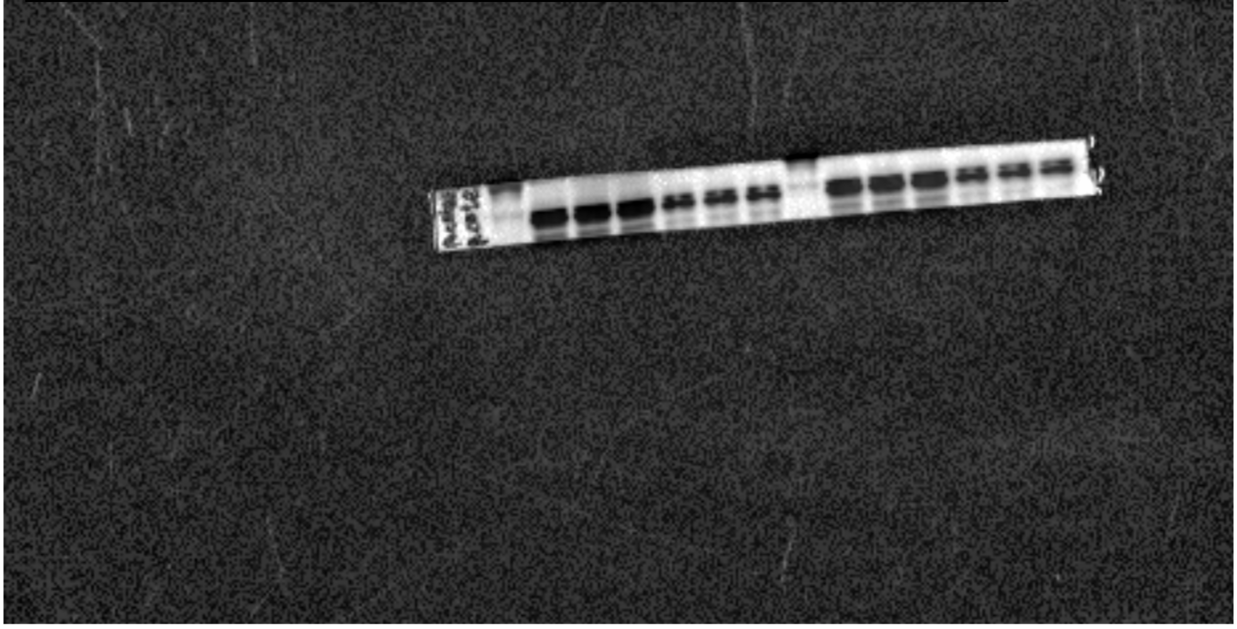

Fig.8: anti-β-actin 42 KDa

Loading sample From left to right:

Three Vector groups, three NTN4 groups (786-O)

Three Vector groups, three NTN4 groups (769-P)

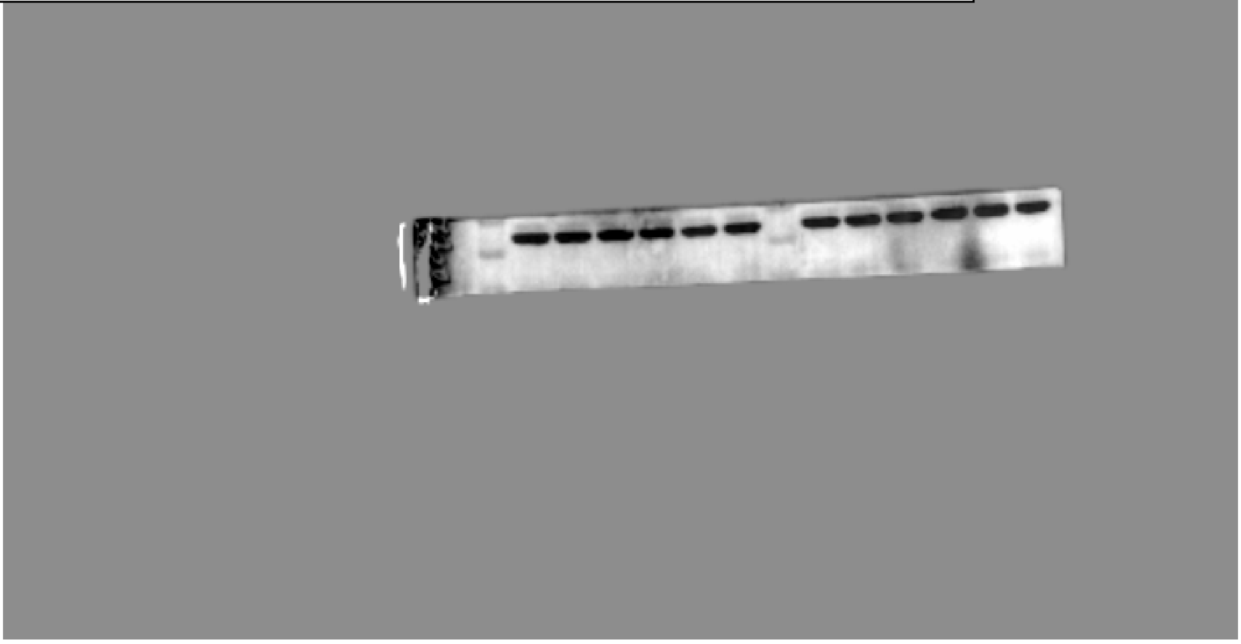

Fig.8: anti-β-catenin 92 KDa

Loading sample From left to right:

Three Vector groups, three NTN4 groups (786-O)

Three Vector groups, three NTN4 groups (769-P)

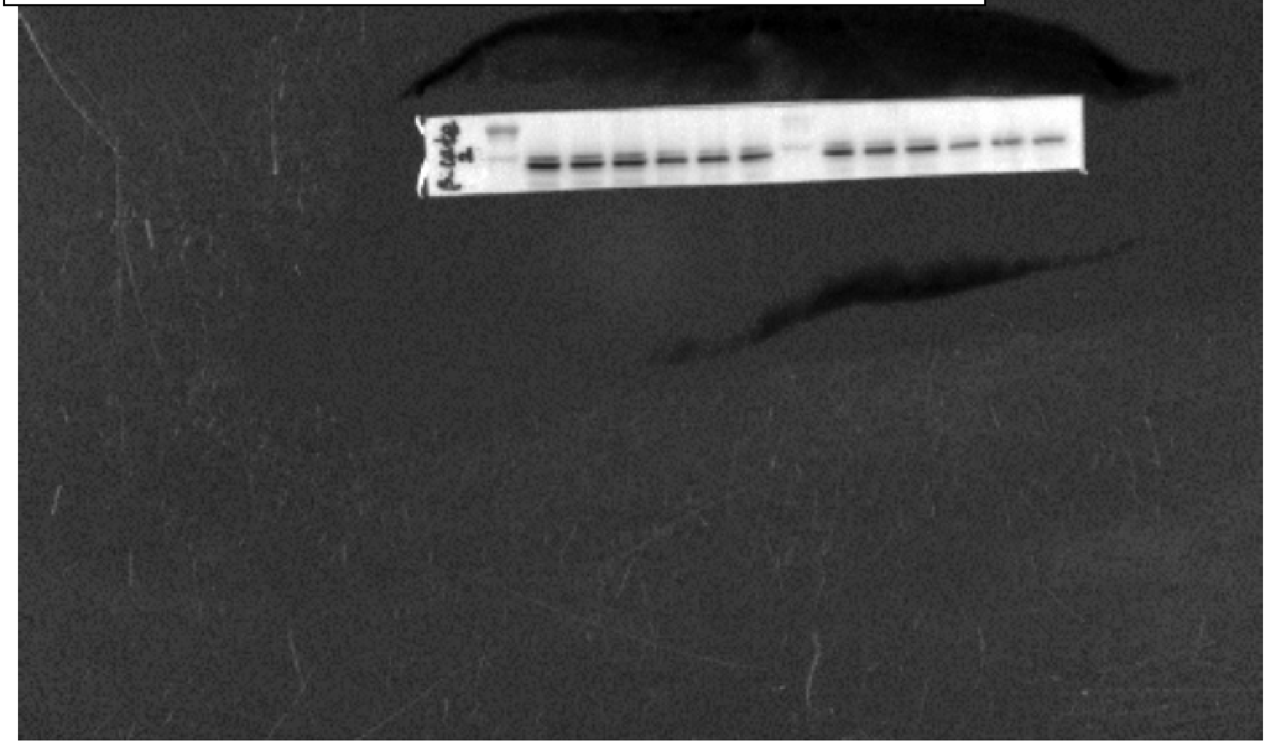

Fig.8: anti-laminB 66 KDa

Loading sample From left to right:

Three Vector groups, three NTN4 groups (786-O)

Three Vector groups, three NTN4 groups (769-P)

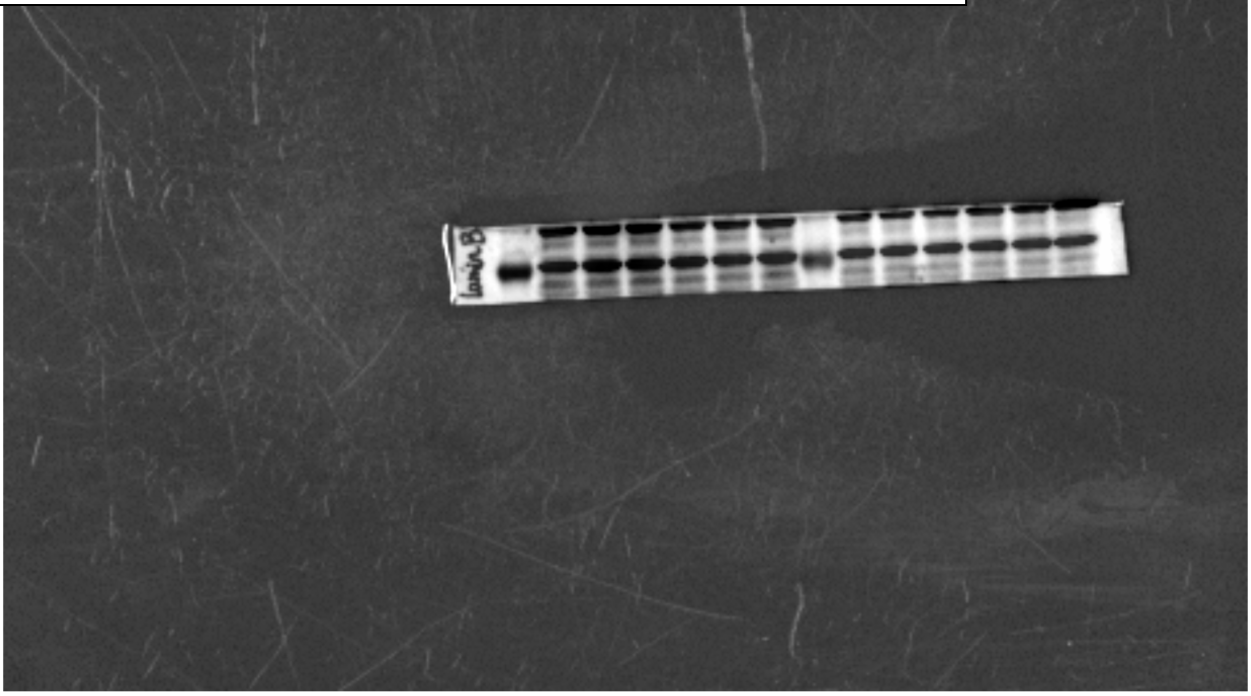

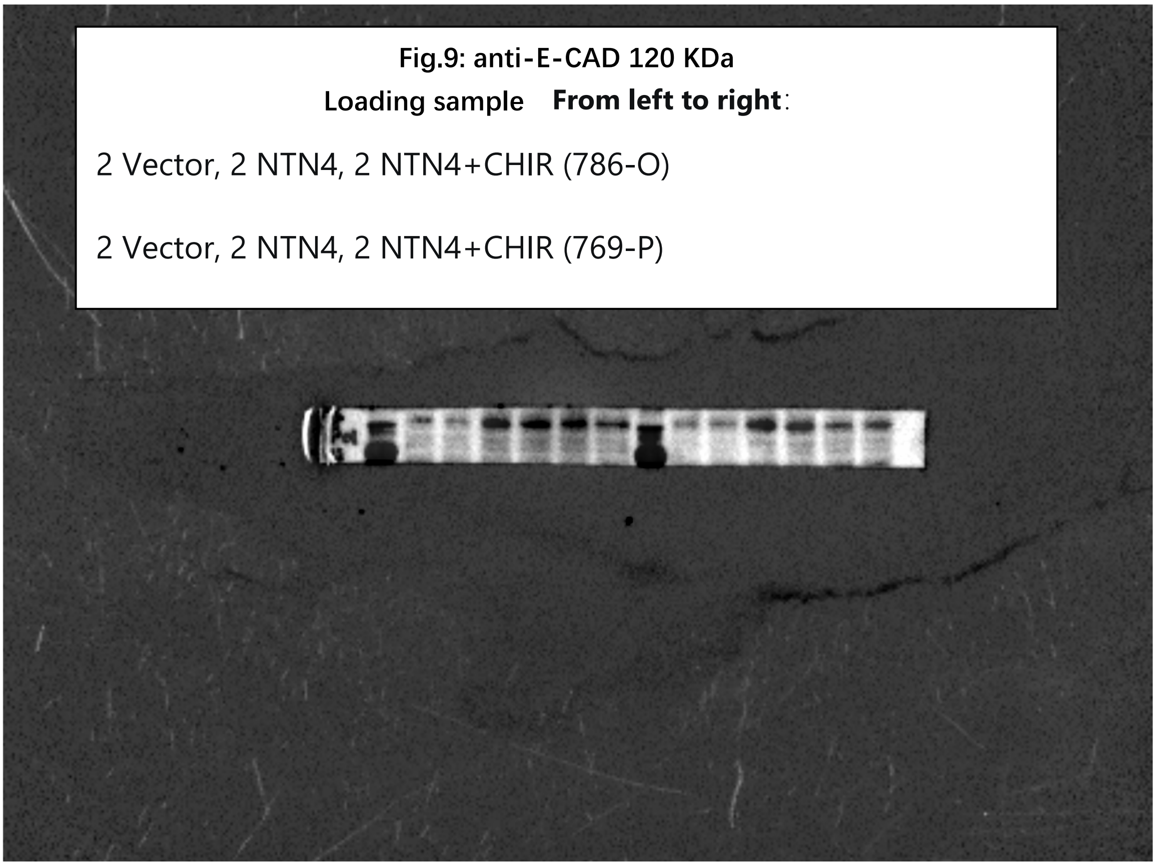
Fig.9: anti-E-CAD 120 KDa
Loading sample **From left to right**:
2 Vector, 2 NTN4, 2 NTN4+CHIR (786-O)
2 Vector, 2 NTN4, 2 NTN4+CHIR (769-P)

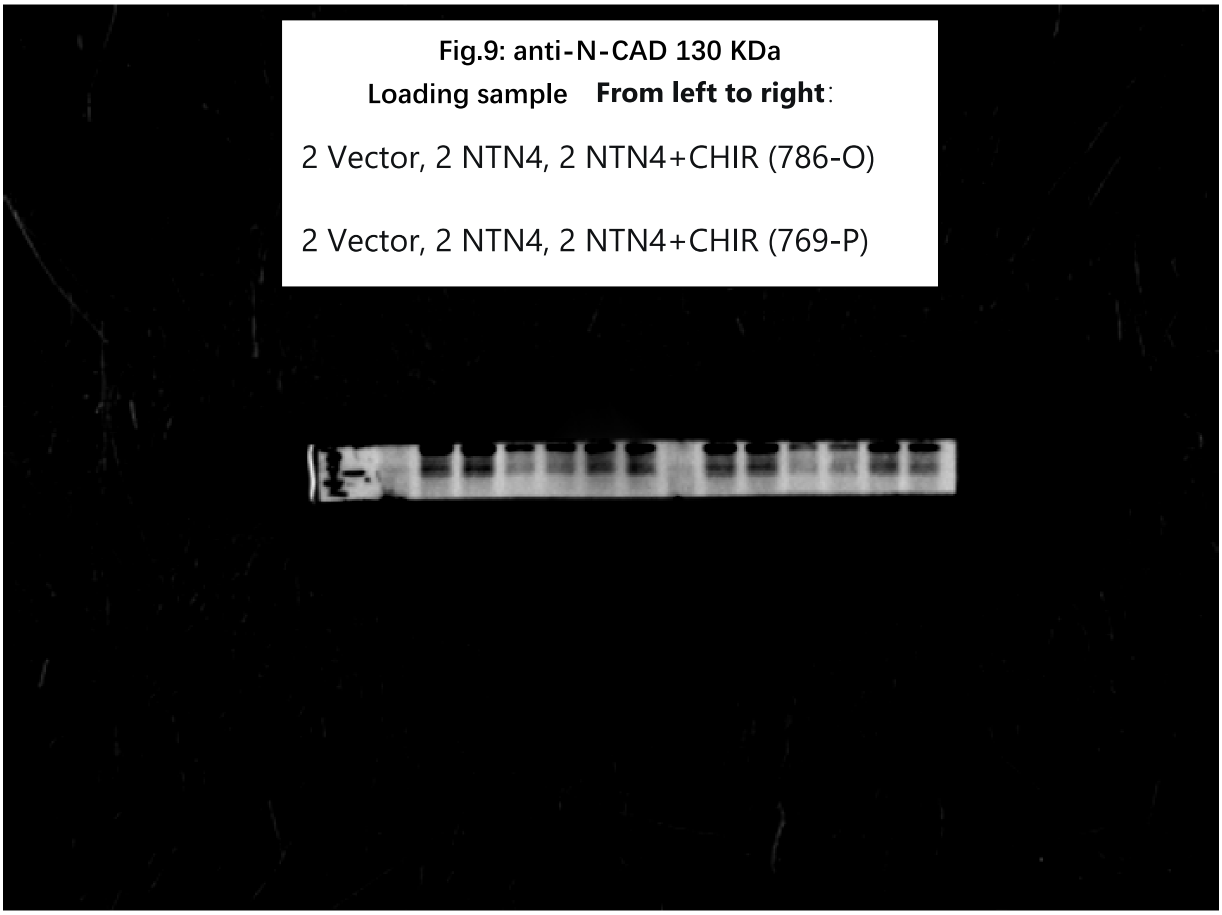
Fig.9: anti-N-CAD 130 KDa
Loading sample **From left to right**:
2 Vector, 2 NTN4, 2 NTN4+CHIR (786-O)
2 Vector, 2 NTN4, 2 NTN4+CHIR (769-P)

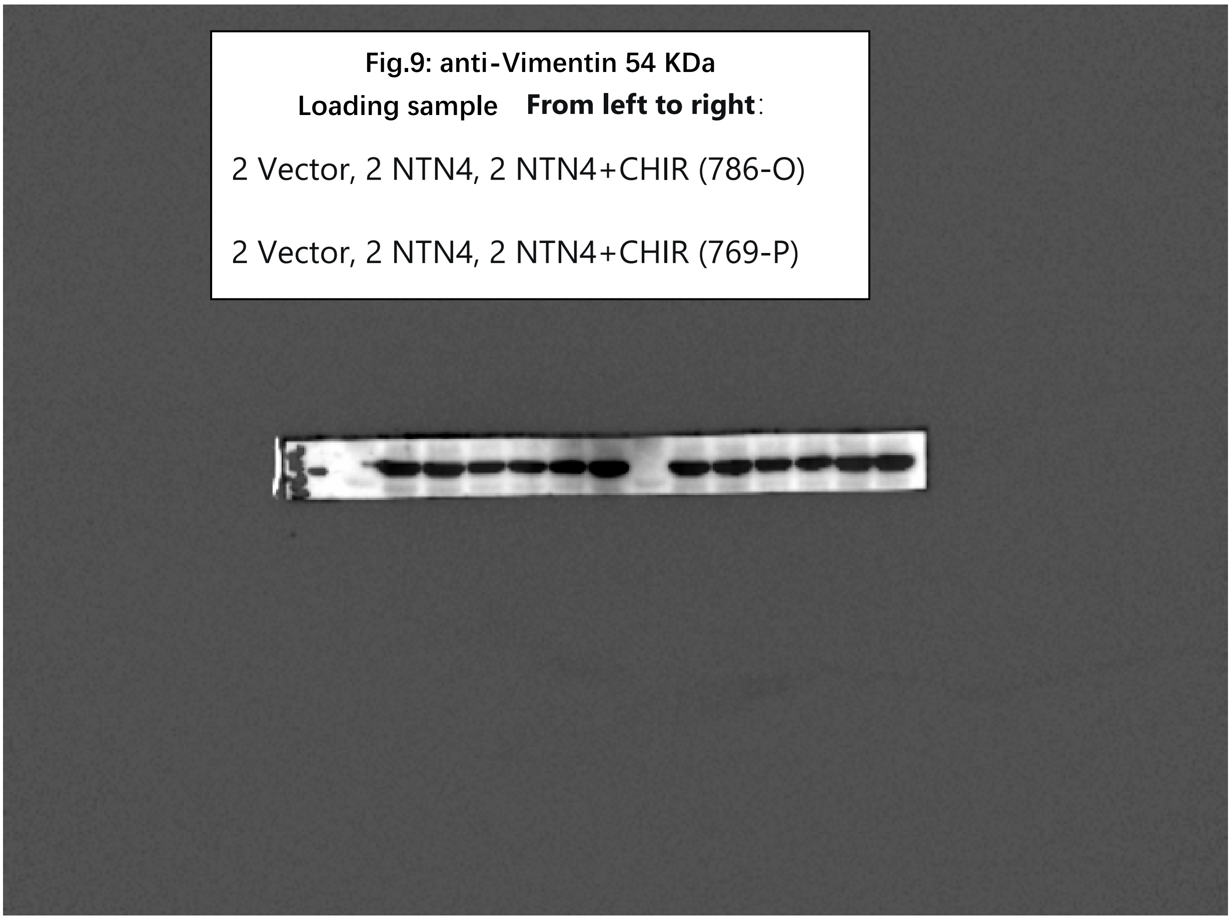
Fig.9: anti-Vimentin 54 KDa
Loading sample From left to right:
2 Vector, 2 NTN4, 2 NTN4+CHIR (786-O)
2 Vector, 2 NTN4, 2 NTN4+CHIR (769-P)

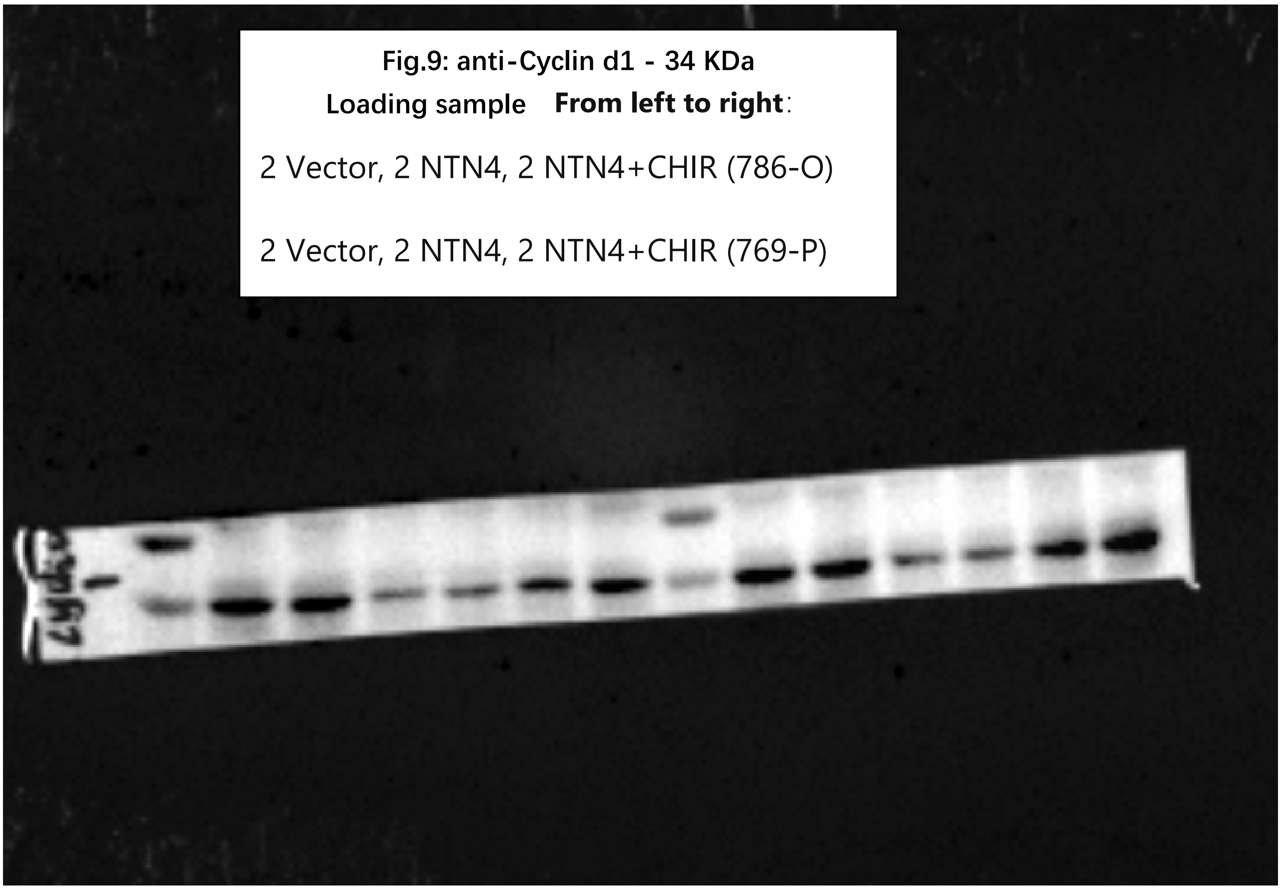
Fig.9: anti-Cyclin d1 - 34 KDa
Loading sample From left to right:
2 Vector, 2 NTN4, 2 NTN4+CHIR (786-O)
2 Vector, 2 NTN4, 2 NTN4+CHIR (769-P)

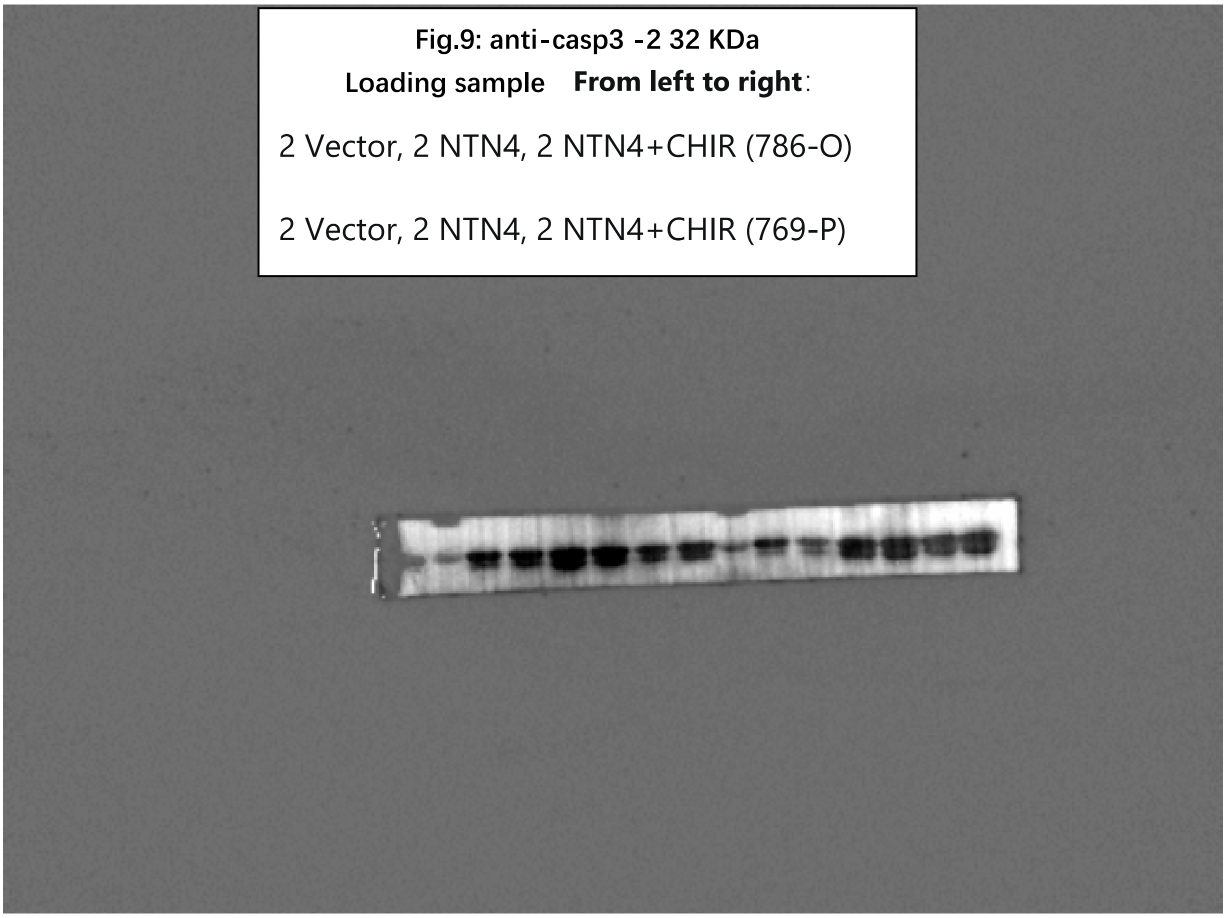
Fig.9: anti-casp3 -2 32 KDa
Loading sample From left to right:
2 Vector, 2 NTN4, 2 NTN4+CHIR (786-O)
2 Vector, 2 NTN4, 2 NTN4+CHIR (769-P)

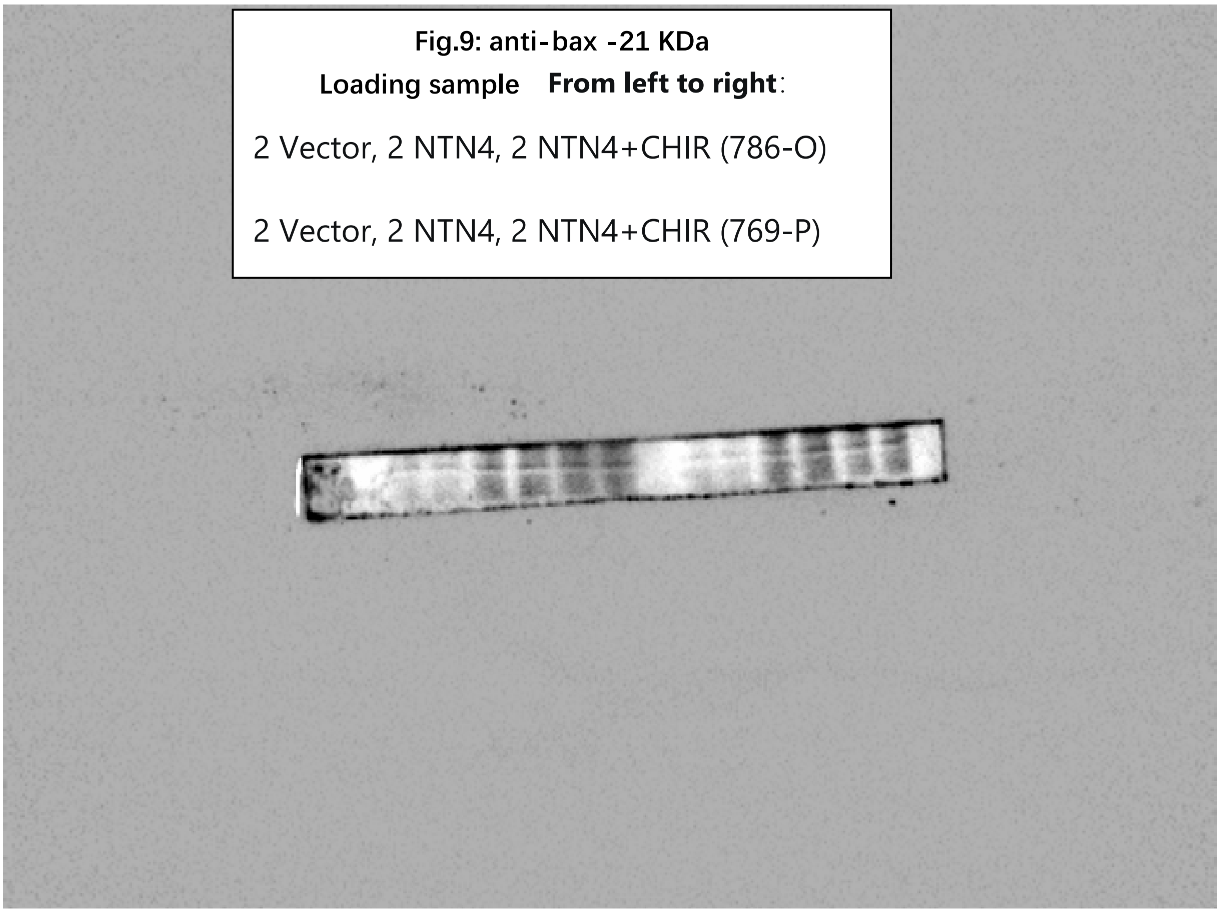
Fig.9: anti-bax -21 KDa
Loading sample From left to right:
2 Vector, 2 NTN4, 2 NTN4+CHIR (786-O)
2 Vector, 2 NTN4, 2 NTN4+CHIR (769-P)

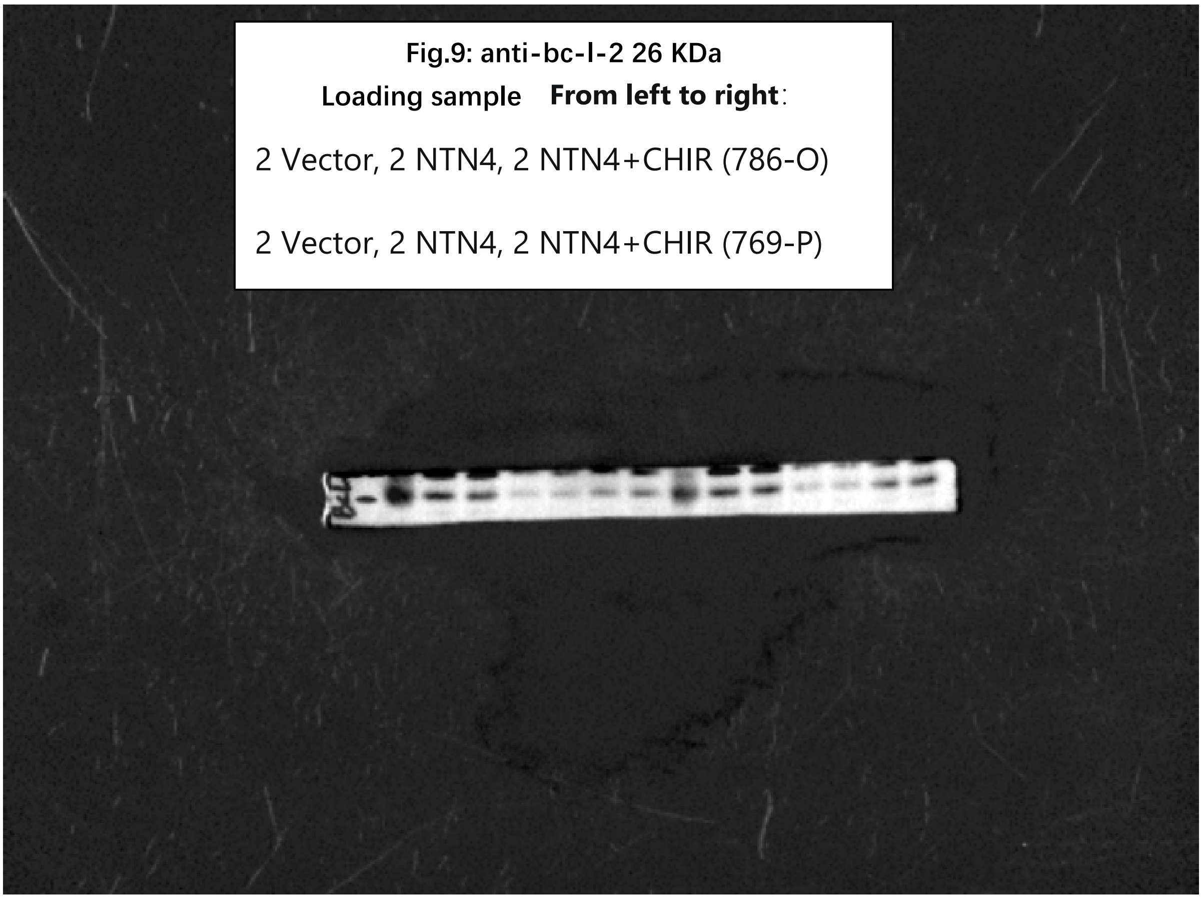

Fig.9: anti-bc-l-2 26 KDa

Loading sample **From left to right**:

2 Vector, 2 NTN4, 2 NTN4+CHIR (786-O)

2 Vector, 2 NTN4, 2 NTN4+CHIR (769-P)

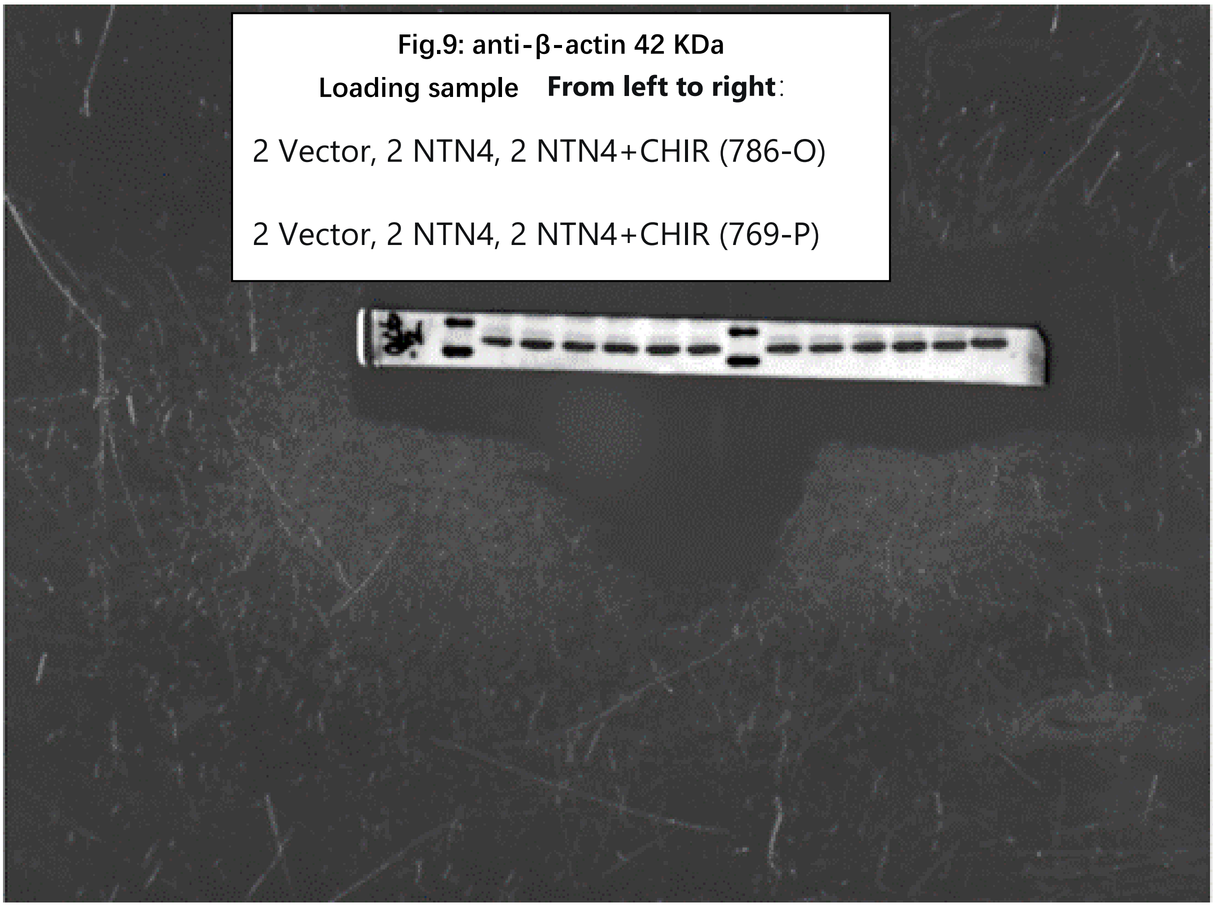

Fig.9: anti-β-actin 42 KDa

Loading sample **From left to right**:

2 Vector, 2 NTN4, 2 NTN4+CHIR (786-O)

2 Vector, 2 NTN4, 2 NTN4+CHIR (769-P)

Fig.10: anti-β-catenin 92 KDa

Loading sample From left to right:

Three Vector groups, three NTN4 groups

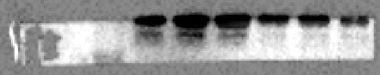

Fig.10: anti-Vimentin 54 KDa

Loading sample From left to right:

Three Vector groups, three NTN4 groups

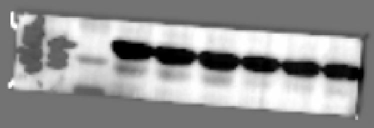

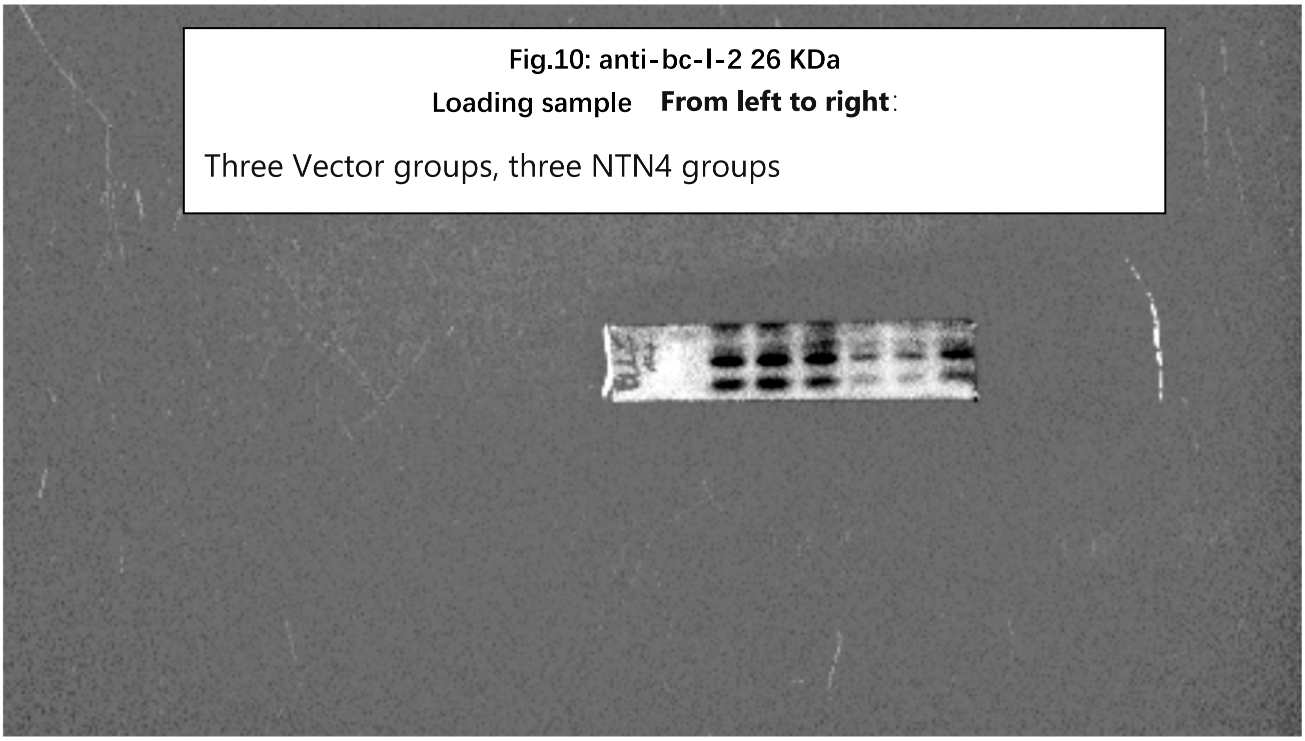

Fig.10: anti-bc-l-2 26 KDa

Loading sample From left to right:

Three Vector groups, three NTN4 groups

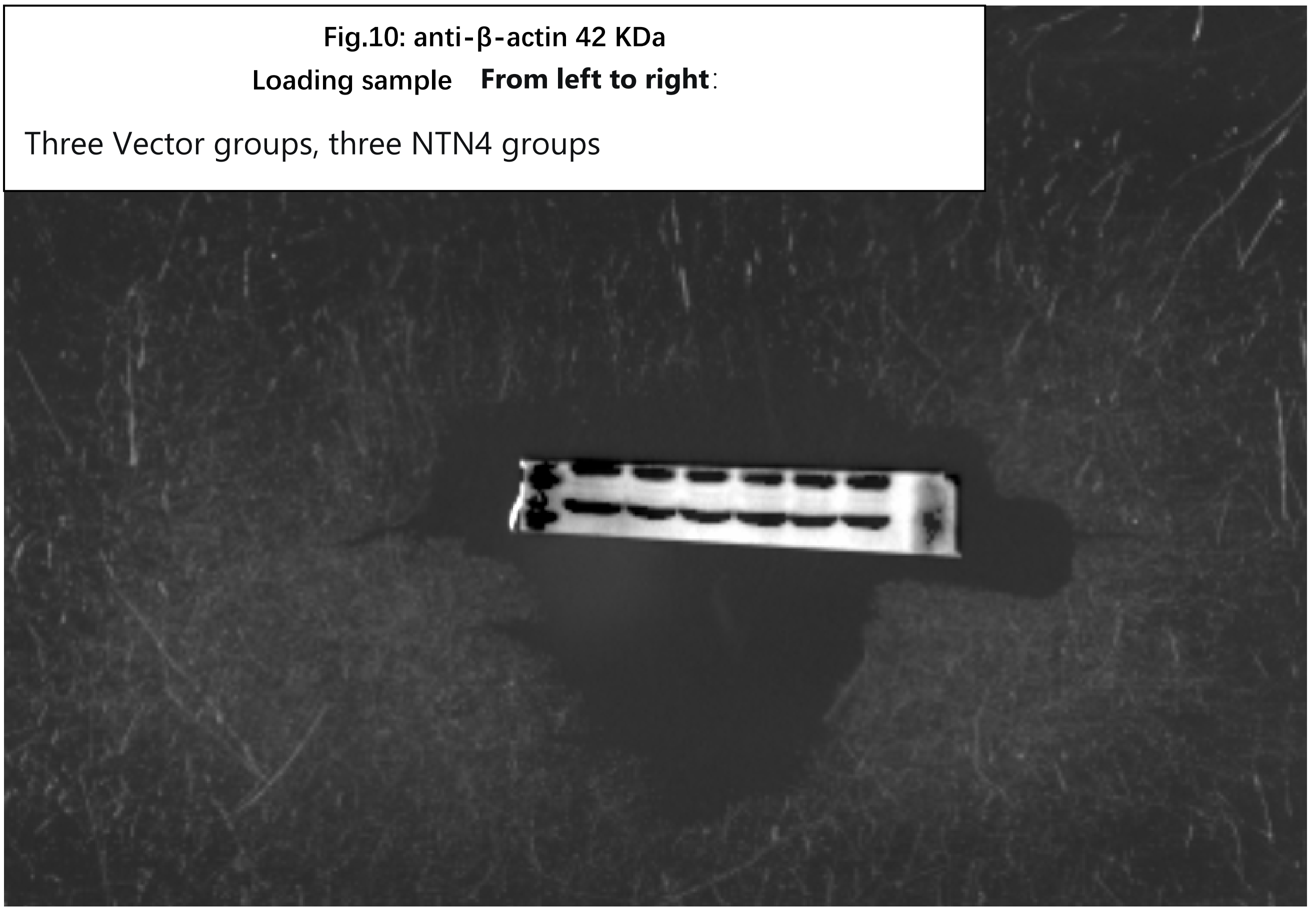

Fig.10: anti-β-actin 42 KDa

Loading sample From left to right:

Three Vector groups, three NTN4 groups
